# Will COVID-19 Lead to Myalgic Encephalomyelitis/Chronic Fatigue Syndrome?

**DOI:** 10.3389/fmed.2020.606824

**Published:** 2021-01-18

**Authors:** Anthony L. Komaroff, Lucinda Bateman

**Affiliations:** ^1^Brigham and Women's Hospital and Harvard Medical School, Boston, MA, United States; ^2^Bateman Horne Center, Salt Lake City, UT, United States

**Keywords:** COVID-19, SARS-CoV-2, post-infectious fatigue syndrome, post-viral fatigue syndrome, myalgic encephalomyelitis/chronic fatigue syndrome

## Introduction

“Recovering” from COVID-19 does not guarantee a return to a person's usual state of health. For one thing, some people with multi-system injury—particularly to the brain, heart and kidneys—may develop permanent dysfunction of those organs.

In addition, a more subtle form of chronic illness may develop. For some people with COVID-19, even those who are only mildly affected at first, the ensuing weeks and months of “recovery” bring a surprise and a betrayal: they do not return to full health. Although nucleic acid tests no longer detect the virus, people still suffer from ongoing symptoms. They call themselves “long haulers,” and the condition is being called “long COVID.”

## How Common Is a Lingering Post-COVID-19 Illness?

The Centers for Disease Control and Prevention (CDC) followed nearly 300 people who were PCR-positive for SARS-CoV-2 for several weeks. Three weeks after the positive test, nearly half of the patients still had symptoms, such as fatigue and cough—particularly people who were older or suffered from chronic diseases (1).

Italian investigators studied 143 confirmed COVID-19 patients after the most severe symptoms had ended. Sixty days after the onset of their illness, more than half of the patients continued to have multiple bothersome symptoms, and 41% reported a worsened quality of life (2).

Irish investigators studied 128 patients with PCR-documented SARS-CoV-2 infection and found that, at a median of 10 weeks after the initial COVID-19 symptoms, 52% reported persistent fatigue and 31% had not returned to work. Surprisingly, there was no association of post-COVID fatigue with the severity of the acute illness, nor with routine laboratory markers of inflammation and cell turnover (3).

Between December, 2019 and May, 2020, a group of patients conducted an online survey of patients who, by self-report, experienced symptoms consistent with COVID-19, in collaboration with University College, London; Weill Cornell Medicine, New York, NY; and Oregon Health and Science University, Portland, Oregon. The survey consisted of 257 questions, was translated from English to eight other languages, and was completed by 3,762 patients (age 18 or older) from 56 countries—predominantly white, middle-class, and English-speaking. Of the respondents, 8.4% reported being hospitalized, and 27% reported a laboratory-confirmed diagnosis. At 7 months after the onset of the illness, continue fatigue, post-exertional malaise and cognitive dysfunction (all core symptoms of ME/CFS) remained in 77.9, 71.2, and 56.8%, and 67.5% were unable to work or required a reduced work schedule compared to prior to the illness onset. Systematic physical examination and laboratory diagnostic panel was not performed (4). The data, as reported, don't allow a determination of how many of these people with possible COVID-19 met criteria for ME/CFS, but it is plausible that the majority did.

## Post-Infectious Fatigue Syndromes

It is not surprising that some people infected with the COVID-19 coronavirus (SARS-CoV-2) develop a debilitating chronic fatigue. Post-infectious fatigue syndromes follow in the wake of acute infections with several different types of infectious agents: viruses, such as SARS coronavirus (5), Epstein-Barr virus (6–8), Ross River virus (8), enteroviruses (9), human herpesvirus-6 (10), Ebola virus (11), West Nile virus (12), Dengue virus (13), and parvovirus (14); bacteria, such as *Borrelia burgdorferi* (15), *Coxiella burnetii* (16), and *Mycoplasma pneumoniae* (17); and even parasites, such as *Giardia lamblia* (18). The *acute* symptoms of these illnesses, and the organ damage they cause, can be very different. However, the lingering chronic fatiguing illness following each illness appears to be quite similar.

## Myalgic Encephalomyelitis/ Chronic Fatigue Syndrome (ME/CFS)

People with post-infectious fatigue syndromes following these well-documented acute infections share a group of symptoms in common with people who have ME/CFS (originally called just “chronic fatigue syndrome”). Many, but not all, people with ME/CFS note that it began suddenly, with an apparently infectious illness characterized by respiratory symptoms, fever, adenopathy, myalgias, and other symptoms. Because such acute illnesses are common and typically resolve, often no attempt has been made to diagnose the inciting infectious agent. Yet the spectrum of symptoms in ME/CFS that follows an apparently infectious illness due to an undocumented infectious agent is very similar to the illness following a well-documented infectious agent.

Indeed, according to Dr. Anthony Fauci, the Director of the National Institute for Allergy and Infectious Diseases, patients post-COVID-19 can develop “a post-viral syndrome that's very strikingly similar to myalgic encephalomyelitis/chronic fatigue syndrome” (19).

A widely-used case definition of ME/CFS was proposed by the U.S. National Academies of Sciences, Engineering and Medicine (NASEM) (20). This case definition requires that the illness must have lasted for at least 6 months. Since most people who have developed COVID-19 in the U.S. have not yet been ill for 6 months, not enough time has elapsed to know how many will develop an illness that meets the case definition of ME/CFS. We think it is likely that some will.

What is the current burden from ME/CFS in the United States? CDC and NASEM estimate that between 836,000 and 2.5 million Americans suffer from ME/CFS; the direct and indirect economic costs of the illness to society are estimated to be between $17 and $24 billion each year (20).

## How Many Additional Cases of ME/CFS Will be Caused by the Pandemic?

One can only guess about the future, but we propose a few conservative estimates. As of late December 2020, nearly 20 million Americans have tested positive for SARS-CoV-2. Based on serological studies the CDC estimates that the true number of infections may be exponentially higher.

To estimate the number of people in the U.S. who may develop “long COVID” we make two conservative assumptions: (1) the introduction of effective vaccines in late 2020 and early 2021 will constrain the total number of people in the U.S. who become infected with SARS-CoV-2 to only 25 million Americans by the end of 2021; and (2) although over 50% of people with confirmed or suspected COVID-19 state that they remain with lingering symptoms at 3 months, we assume that only 10% will be left with an illness that meets the NASEM case definition for ME/CFS. This is consistent with a careful prospective study of the course of symptoms following three quite different acute infections (8). Over the course of 1 year, that would at least double the number of Americans suffering from ME/CFS. The annual incidence of the illness would equal or surpass the point prevalence—a remarkable event in the history of a chronic illness.

What might this mean globally? As of December 2020, COVID-19 has been documented in about 80 million people, globally. Using similar estimates to those we used for the U.S., that number would be predicted to increase to nearly 110 million during 2021, and to generate over 10 million new cases of ME/CFS, globally.

Of course, these number are all rough guesses. But they are informed by well-measured prior experience, and suggest that the U.S. and the world will see a substantial growth in the number of people with ME/CFS. How lasting that illness will be, we cannot know. Most long-term longitudinal studies of people with ME/CFS before the pandemic found that, in most patients, the illness had not abated after many years (21, 22), although the prognosis may be somewhat better in children with ME/CFS (23).

## What Causes Post-Infectious Fatigue Syndromes and ME/CFS?

Any acute infectious disease, like COVID-19, that damages multiple organ systems can cause chronic symptoms (along with objective physiological abnormalities) in some people. The symptom of chronic fatigue could be caused by impaired function of the heart, lung, or kidneys. It is too early in the COVID-19 pandemic to know how many will suffer permanent dysfunction of these organs, but it surely is possible. Therefore, in some people with persistent, debilitating fatigue following COVID-19, documentable damage of these organs may be a sufficient explanation of their fatigue. Careful longitudinal studies assessing both symptoms and physiologic function will be necessary to know whether, and how often, this happens.

Experiencing a life-threatening illness, particularly when extreme measures, such as artificial ventilation are required, can lead to post-traumatic stress disorder (PTSD). And if a patient has not been able to return to pre-illness function due to chronic symptoms, the persistent symptoms may also trigger major depression. These psychiatric disorders also may lead to chronic fatigue and related symptoms.

Yet, many cases of post-infectious fatigue follow in the wake of acute infections that are not known to cause permanent damage to the heart, lungs or kidneys—and in people without comorbid PTSD or depression. In the typical case of ME/CFS, in particular, the inciting “infectious-like” illness most often appears to be a transient infection, or a primary infection that becomes permanent but does not typically produce chronic organ dysfunction (such as occurs with Epstein-Barr virus).

What might explain the fatigue and other symptoms if there is no documented heart, lung or kidney damage? Although uncertain, it is likely that the causes of all post-infectious fatigue syndromes share with each other and with ME/CFS many common elements (24). Longitudinal studies of people who develop COVID-19 may help reveal the biological underpinnings of many post-infectious fatigue syndromes.

In people with lingering fatigue post-COVID-19—and without chronic cardiac, pulmonary or renal dysfunction—one likely explanation for the chronic fatigue is a state of chronic low-grade neuroinflammation generated by the disease (25).

SARS-CoV-2 can infect the brain, causing neuroinflammation (26). Moreover, inflammation elsewhere in the body can activate the innate immune system in the brain via both humoral and retrograde neural signals, largely involving the vagus nerve (27, 28). As argued elsewhere (24), neuroinflammation can produce fatigue through the action of various cytokines, perhaps acting on a “fatigue nucleus”—a collection of neurons dedicated to diminishing energy-consuming activities (“sickness behavior”). Such energy-conserving behavior in an organism that is infected or injured would help to focus available energy stores on the process of healing (27, 29). In addition to activation of a “fatigue nucleus” by neuroinflammation, a state of chronic, severe fatigue and related symptoms could also be explained by other abnormalities identified in ME/CFS: impaired energy production (30), oxidative stress (31), ion channelopathies (32), and impaired cerebral perfusion (33).

The longitudinal studies that need to be conducted include repeatedly collecting information on the presence and severity of various symptoms—symptoms common in people with COVID-19 and symptoms that are part of case definitions of ME/CFS. Such studies also should include repeated laboratory studies of the immune system, metabolism, gene structure, and the transcriptome, as well as tests of thinking, sleep, and the functioning of the nervous system, heart, and cardiovascular system.

## What Are the Implications for Practicing Physicians?

Although there now is a literature of over 9,000 peer-reviewed studies of ME/CFS, as identified by NASEM (20), it is our experience that many practicing physicians know little about the illness. If a wave of new cases that doubles the number of Americans with ME/CFS is about to emerge, we need to increase efforts to prepare physicians to deal with this burden. A U.S. ME/CFS Clinician Coalition—physicians experienced in the care of people with ME/CFS—has created a website containing useful information^1^. CDC^2^ and NIH^3^ also provide online information.

## Conclusion

The COVID-19 pandemic has been a tragedy. It has devastated the health and financial well-being of many people around the world. An unprecedented effort is underway to understand, prevent and treat the disease, including substantial recent funding to study post-COVID illnesses, in the U.S. and elsewhere.

We should not forget the importance of studying all people who become infected with SARS-CoV-2, even those with only mild initial illnesses, and to study the recovery period and the long-term health consequences of COVID-19. We need to know how to prevent and treat “long COVID.” What we learn may apply to the prevention and treatment of ME/CFS, as well.

## Author Contributions

AK conceptualized the paper and wrote the original draft. AK and LB reviewed and finalized the paper. All authors contributed to the article and approved the submitted version.

## Conflict of Interest

AK reports receiving personal fees from Serimmune Inc., Ono Pharma, and Deallus, and grants from the National Institutes of Health, for activities unrelated to the submitted work. LB is employed by the Bateman Horne Center which receives grants from the National Institutes of Health, and from BHC receives fees from Exagen, Inc., and Teva Pharmaceutical for activities unrelated to the submitted work.

## Footnotes

^1^MECFSClinicianCoalition.org

^2^https://www.cdc.gov/me-cfs

^3^https://www.nih.gov/mecfs/about-mecfs
